# Multiple Sclerosis State of the Art (SMART): A Qualitative and Quantitative Analysis of Therapy's Adherence, Hospital Reliability's Perception, and Services Provided Quality

**DOI:** 10.1155/2014/752318

**Published:** 2014-08-26

**Authors:** G. Di Battista, A. Bertolotto, C. Gasperini, A. Ghezzi, D. Maimone, C. Solaro

**Affiliations:** ^1^Neurology Unit, Department of Neuroscience and Sense Organs, A.C.O San Filippo Neri, Via G. Martinotti 20, 00135 Rome, Italy; ^2^Clinical Neurobiology Unit, Regional Referring Multiple Sclerosis Centre (CReSM), University Hospital San Luigi Gonzaga, Orbassano, 10043 Turin, Italy; ^3^Department of Neurology, A.O. San Camillo Forlanini Hospital, Circonvallazione Gianicolense 87, 00152 Rome, Italy; ^4^Multiple Sclerosis Study Centre, Gallarate Hospital, Via Pastori 4, 21013 Gallarate, Italy; ^5^Neurology Unit, A.O. Garibaldi Nesima, Piazza Santa Maria Del Gesù 5, 95122 Catania, Catania, Italy; ^6^Neurology Unit, Department Head and Neck, ASL3 Genovese, Via D. Oliva 22, 16154 Genoa, Italy

## Abstract

The purpose of this study was to assess the adherence to therapy in patients with relapsing remitting multiple sclerosis (RR-MS) and to analyze the possible influence of factors such as hospital care and patients socioeconomic status. Two hundred eighty-five patients with RR-MS according to Mc Donald's criteria and naïve disease-modifying drugs (DMDs) naïve were enrolled. Two self-administered questionnaires addressing the management of patients at therapy prescription and the personal perception of the daily life changes caused by DMDs were administered at months 3 and 12. Full adherence, considered as correct use of the therapy prescribed, was observed in a very high percentage of subjects (97.3% and 93.9% at 3 and 12 months). The main cause for reduced adherence was single dose forgetfulness, followed by anxiety, pain at the injection site, and tiredness of “doing all injections.” Nurses and neurologists of MS Center were identified as the major resource in coping with the disease at 3 and 12 months by patients. The neurologist was the health professional involved in MS management in 95% of cases and the nurse appeared to play a central role in patient training and drug administration management (50.3%).

## 1. Background and Objective

Adherence to prescribed treatment in chronic disease is a critical factor for a successful therapeutic response; however, in conditions like multiple sclerosis (MS), where the treatment is mainly preventive, it may be inadequate. The reasons for poor adherence may be related to lack of perception of immediate benefits and to the inconvenience and discomfort of injectable treatment.

The general definition of treatment adherence [1] includes treatment persistence and compliance. Treatment persistence refers to a patient's enduring motivation to continue a given treatment and it can be measured by the time from initiation to discontinuation of therapy. In the World Health Organization [2] project for long-term therapy, adherence (treatment compliance, synonym: adherence) has been defined as “the extent to which a person's behaviour—taking medication, following a diet, and/or executing lifestyles changes—corresponds to agreed recommendations from a healthcare provider” [3] and in patients with MS or other chronicle illness means the extent to which a patient acts in accordance with the prescribed interval and dosing of a drug regimen. The compliance is measured over a period of time and reported as a percentage.

There are not many studies on adherence in MS and most of them mainly focus on discontinuation of therapy [4–6] rather than adherence intended as proper use of therapy according to prescription (number of missing doses) [7]. The reason for failing in assessing adherence may derive from to the difficulty in monitoring the data [8].

Noncompliance is frequent among patients with MS taking disease-modifying drugs (DMDs) because they sometimes forget a dose or deliberately withhold it. In the Global Adherence Project, Devonshire and colleagues [9] found that the most common reason for noncompliance among patients with MS was forgetting to inject (50%). Anecdotally, patients frequently cite other reasons for noncompliance, such as fatigue, needing a break, or adverse events.

There are several causes that may lead to suspension or irregular management of therapy including occurrence of side effects which worsen the quality of life, perceptions of ineffective or unnecessary therapy, forgetfulness, and incorrect understanding of the drug. Furthermore, subjects treated in clinical practice may receive a less intensive follow-up by specialist practitioners than those included in clinical trials, and this can further interfere negatively in adherence and efficacy [10–12]. Mohr et al. [13] reported also that the therapy is continued regularly in 86% of patients with depression if treated with antidepressants, whereas in untreated depressed patients adherence falls to 38%.

The purpose of this study was to assess the treatment adherence to DMDs, intended as adherence to prescribed number of doses and doses voluntarily not taken without permission, in patients with relapsing remitting MS (RR-MS). The study also included the analysis of possible factors such as hospital care and patients socioeconomic status.

## 2. Materials and Methods

SMART (State of the Art Multiple Sclerosis) is a prospective observational multicentric study using self-administered questionnaires conducted in 34 MS centers at public hospitals, distributed throughout the national territory and authorized to prescribe DMDs for MS. The questionnaires included the following items and were administered 3 months and 1 year after drug prescription.

*Items Reported in the Questionnaires*. Demographic and socioeconomic data include

- sex;
- age (years);
- what your qualification is;
- what your occupation is;
- who currently lives.

MS Diagnosis and management include

- age at diagnosis of MS (only 3 months);
- whether you are currently followed up at the center where you were first diagnosed;
- which health care professional regularly sees your condition;
- how often on average sees your neurologist;
- how often medium sees your neurologist;
- how long you are engaged in the consultation with a neurologist (min);
- how often you contact your nurse;
- how long you engaged in a visit with the nurse (min).

Which of the following factors is important in the choice of therapy for the MS?

- The drug's mechanism of action;
- The drug reduces the relapses;
- The drug slows the progression of the disease;
- The drug produces fewer antibodies;
- The drug is well known;
- The drug has few side effects;
- The drug improves my MRI;
- The support of a nurse to give injections;
- The information support provided by the company that makes the drug;
- The mode of administration (im/sc);
- The ability to self-administer the injections;
- The availability of an autoinjector;
- How many times a week I should take the drug;
- The independence that a treatment can give.

Who prepared you for taking the medication?

- Was I already aware of these treatments for MS? (only 3 months).
- Was I prepared to give myself injections? (only 3 months).
- By whom was I prepared? (only 3 months).
- Who administers the injections? (only 3 months).
- What is the main resource in dealing with the disease?

Therapy includes the following.

- Who decided the treatment? (only 3 months).

Judgment on therapy for MS includes the following.

- Do you feel satisfied with the current treatment?
- What do you think of the current therapy?
- Do you believe the therapy can slow the progression of the disease?
- Please indicate how much you agree or disagree with what is written below.
- What are the side effects of treatment?
- My symptoms improved a year ago (only 12 months).
- How many times a week according to your neurologist's prescription do you make the injections?
- How often do you apply the regimen prescribed by your neurologist?
- Would you tell your neurologist that you did not follow perfectly what he prescribed?
- How many injections have you forgotten by choice or by accident in the last 4 weeks?
- How many injections have you missed?
- Have you decided to change the dose to inject in the last 4 weeks?

Eligible patients were identified at the time of first therapy prescription. Inclusion criteria were age older than 18 years, being diagnosed with RR-MS according to the 2005 revised Mc Donald's criteria [14], and being naïve to therapy with DMDs at the time of study entry. Furthermore, to participate in the study, patients should agree to start therapy, sign informed consent, and fill out the first diary to 3 months.

After having signed the informed consent, each patient received and filled the questionnaires. A medical form including demographic and clinical data was also filled out at enrollment and updated during the follow up.

All patients began treatment immediately after the screening with EMA-approved DMDs (IM interferon beta-1a [Avonex, Biogen Idec], interferon beta-1b [Betaferon, Bayer], glatiramer acetate [Copaxone, Teva Neuroscience], and SC interferon beta-1a [Rebif, Merck Serono]).

Follow-up duration was 12 months. Two self-administered questionnaires at 3 and 12 months were used to assess adherence to therapy. Furthermore, the first questionnaire evaluated also the management of patients at therapy prescription, whereas the second aimed also to verify the personal perception of the daily life changes caused by DMDs (see the items reported in the questionnaires).

The primary endpoint was the assessment of the treatment adherence considered as acceptance of the prescription made by the physician and analysis of the causes. The secondary endpoint was the identification of the reasons that led to the acceptance, or modification of therapy.

The study was approved by local ethics committee at each center and was conducted according to GCP rules.

## 3. Statistical Analysis

Data were presented as mean ± standard deviation (SD), median, or percent, where appropriate. Statistics were performed by analysis of variance (ANOVA) for continuous variables with normal distribution and by Kruskall-Wallis nonparametric test for discrete variables. Variables of nominal type were analyzed by *χ* ^2^ test and the McNemar test in order to compare the observed frequencies at 3 and 12 months. The duration of treatment was evaluated according to Kaplan-Meier survival curve and Cox model [15–17].

## 4. Results

### 4.1. Patients Characteristics (Table 1)

Of the 285 patients included, 198 were females (69.5%); the mean age was 36 (10.9 SD, Min 16.6, Median 35.4, Max 61.6); and the mean length of education was 12.29 years (SD 3.04, Min 5.0, Median 13.0, Max 20.0). The mean age at MS onset was 31.22 (SD 9.39) and the mean time from onset to diagnosis was 2 years. One hundred sixty out of 263 were working in full or partial time (men 72.2%, women 56.0% reaching 74.5% by adding the housewives), whereas the remaining patients were students (9.5%), retired (4.2%), or unemployed (12.5%). There was no significant loss of employment at 3 and 12 months (McNemar test).

Most of the patients lived at home with their parents or spouse and only 7% lived alone (F 8.0%, M 5.1%).

The mean interval from diagnosis to therapy onset was 1.94 years (SD 3.9) and the EDSS score at study entry was between 0 and 3.5 in 95% and between 4 and 5.5 in the remaining 5% of patients.

Follow-up data were available for 262 patients after 3 months and for 248 patients after 12 months. Thirteen patients discontinued therapy between the first and the second questionnaire and one was lost to follow-up. Causes for treatment discontinuation were ongoing or planned pregnancy (4 cases), refusal of therapy (1 case), comorbidities not related to therapy (2 cases), and side effects due to therapy (depression, elevation of transaminases, and allergy) (1 case).

### 4.2. Therapy's Adherence

A very high percentage of subjects “always try to follow the schedule prescribed” (255/262 pts, 97.3%, at 3 months, and 233/248 pts, 93.9%, and at 12 months; 226 patients (96.6%) out of 234 at both 3 and 12 months confirmed the same response) (Table 6).

The main causes of lack of adherence (Table 7) were forgetfulness of a single dose (14.5% and 17.4% at 3 and 12 months), followed by anxiety generated by injection (12.0% and 13.0% at 3 months and 12 months), pain at the injection site (10.3% at 3 months and 10.4% at 12 months), and tiredness of “doing all injections” (9.4% at 3 months and 7.8% at 12 months).

When asked whether they had missed, by choice or accident, an injection in the past 4 weeks, patients gave negative response in 85.9% and 80.2% of cases at 3 and 12 months, respectively (Table 9). The adherence data are confirmed in Table 10 with 97.2% of patients at 3 months and 95.8% at 12 months stating not to have changed, in the last 4 weeks, the dose prescribed.

Patients concealed their lack of adherence only in 15.3% of cases at 3 months and in 9.7% of cases at 12 months (Table 8). Women responded more sincerely then man, at 3-month questionnaires, with a statistically significant difference in the *χ* ^2^ test with continuity correction (23.7% of men say “no” compared to 12.2% of women, *P* = 0.03). A 12-month (“no” = 9.7%) versus 3-month (“no” = 15.3%) statistically significant difference (*P* = 0.04) was also detectable with McNemar test with continuity correction.

People forgetting or missing the injections also indicates the number of skipped injections: 35 missing doses (mean 2.9 ± 4.1) per person at month 3; 37 missing doses (mean 2.4 ± 1.9) per person at month 12.

### 4.3. Leading Figures in the Management of the Disease

More than 80% of patients affirmed to be followed up at the center where they were diagnosed with MS both at 3- and 12-month evaluation (82% and 87%, resp.), with no gender differences (McNemar test with continuity correction). The neurologist was the health professional involved in MS management in 95% of cases (Table 2).

Neurologists and nurses of MS Center were identified by the patients as the major resource in coping with the disease at 3 months by the patients (for 67.2% and 63.7% of patients, resp.); these data were confirmed at 12 months (for 62.5% and 61.7% of patients, resp.) (Table 3). However, patients believed that the family is the most important resource in coping with the disease burden (81.7% at 3 months, 82.3% at 12 months) (Table 3).

The nurse was identified as the central player in patient training and drug administration management (50.3%) at 3 months followed by the neurologist.

### 4.4. Shared Decision Making and Involvement with Referral Center

The relationship between the people involved in the decision of initiating MS treatment is reported in Table 4. In most cases, the neurologist played a crucial role in making the decision to start DMD therapy, although patients often, more or less actively, participated to this choice (totally 76% of cases). In a very few cases (3/255 pts), patients decided independently from their neurologist and 58/255 patients left the decision to the neurologist.

### 4.5. Factors Influencing the Treatment Choice

The factors influencing the choice of treatment (Table 5) can be clustered in two groups: the drug therapeutic properties and the effects DMDs on daily life. The belief that therapy can prevent relapses or slow the progression of the disease is important for 64.1% and 67.2% of patients, respectively, at 3 months and for 64.3% and 65.6% of patients, respectively, at 12 months.

The presence of side effects is important in 51.4% at 3 months and 44.8% at 12 months. Perceived lack of efficacy and side effects are usually considered the main responsible factors for the discontinuation of therapy [4, 5].

Self-administration of DMDs and the notion that treatment will help the patient to remain independent are the most important factors in determining the therapy choice (53.7% and 59.8% at 3 months and 53.9% and 52.3% at 12 months, resp.) (Table 5). These factors were more relevant in women (64.5%) with statistically significant difference (*P* = 0.03) compared to men (48.7%) at 3 months; the gender difference was persistent, but not statistically significant at 12 months (men 41.7%, women 56.8%).

## 5. Conclusion

The World Health Organization indicates that patients of developed countries with chronic diseases exhibit a therapy adherence of only 50% (1) but assessing the adherence to DMDs in patients with MS is still an unsolved issue. At the present time, there are no reliable markers to verify the adherence to IFN*β* or glatiramer acetate treatment although biological measurement in IFN*β* treated patients can identify a subset of nonadherent patients [18]. An indirect method may be that of requesting patients to return empty vials to be counted, but even in this case there is no certainty whether the data may be accurate or not. The use of electronic devices with recording dose history certainly facilitates the task and provides useful data on forgetfulness but does not exclude the possibility of a voluntary nonadherence.

The overall level of adherence in previous studies was lower than that observed in our cohort [1–3, 7, 19]. In the study of Sabatè [3] on 2314 patients with clinically isolated syndrome (CIS), about 40% of these patients discontinued their first DMT during the observation period. In another study on 97 RRMS patients, Tremlett et al. [7] reported a percentage of 73% of DMDs missed doses, with 1 patient out of 10 having not taken more than 10 doses over a period of 6 months. In this study, the most important factor for missing doses was the amount of alcohol consumed, whereas a low level of educations and the occurrence of relapses were associated with stopping the current DMDs in more than a quarter of the patients. These authors also found that the evidence of missed doses predicted future lack of adherence. No cognitive or psychological test provided useful information. Treadaway et al. [19] used online questionnaires to analyze 798 patients and reported nonadherence rates (missing one or more injections within the last 4 weeks) ranging between 36 and 39%. The most common reason for missing injections was forgetfulness (58%). Other factors including injection-site reactions, quality of life, patients' perceptions on the injectable medications, hope, depression, satisfaction, and support were also assessed in relation to adherence, emphasising the relevance of factors related to the injections procedure itself (site reactions, pain, anticipation anxiety, and difficulty with administration route).

One of the most frequently reported causes of reduced adherence was the difficulty to accept injection therapy (via self-injection). Common reactions were fear, avoidance, anxiety, autonomic reactions, and disgust. Some patients were helped to inject the drug by family members but this behavior can decrease patient's independence and the likelihood of missing injections if the designated family member is not available [20]. In addition to needle phobia, other reasons for skipping doses were the belief that injections are dangerous, that they remind of the status of being ill, and that they may alter the physical aspect [21, 22]. Indeed, the belief that the prescribed therapy can be harmful and that it is a symbol of the disease may be important in altering the adherence even with oral therapies recently introduced in the treatment of MS.

Our study was analysed in the first year of therapy and focused only on adherence to the therapy as proper use of the drug (i.e., taking the medication at the right time and dose, on the right day). Previous studies [7, 23] already focused on the acceptance of therapy, allowing a prediction on the possible adherence mainly in the initial phase of therapy. In subsequent periods, other factors such as the perception of the effectiveness of therapy may prevail [24] and assessments of adherence in the long term can be mainly analyzed retrospectively.

The percentage of adherence observed in our prospective study (97.3% and 93.9% at 3 and 12 months) was higher than those previously reported (generally not greater than 70%). This finding is probably related to the active role played by hospital neurologists and nurses, who were directly involved to share the decision of treatment initiation and substantially contributed to educate patients to properly administer the drug, cope with adverse events, and plan the clinical follow-up. Meyniel et al. [4] report indeed discontinuation rates for DMDs greater in Canada (51.4%) and Australia (47.3%) than in Italy (38.1%) or Spain (29.4%).

Our interpretation is supported by the response to the question “Decide to modify the prescribed dose in the last 4 weeks?” (Table 10) with a negative response in 97.2% at 3 months and 95.8% at 12 months but is partially weakened by answer to the question “Would you tell your neurologist you did not follow his prescriptions?” (Table 7), where 15.7% at 3 months and 9.7% at 12 months claimed not to tell their nonadherence to the neurologist. To the question “Have you forgotten, by choice or by accident, a few injections in the last 4 weeks?” (Table 9), the 85.9% at 3 months and 80.2% at 12 months of patients provide a negative response. To our opinion, these data underlie the intention of being adherent to therapy, despite a few doses missed, as this seemed to happen occasionally and not deliberately (simple forgetfulness or other occasional factor).

Equally important was the analysis of interactions in the starting therapy decision as a patient's “active,” “passive,” or “collaborative” [20] participation in disease management (Table 4) might had an impact on adherence. Shared decision making or shared opinion was the behavior of choice in 1/3 of our patients, but is increasingly recognized as the ideal model of patient-physician communication especially in chronic diseases, such as MS, with partially effective treatments as MS.

MS centres are recognized to have a central role in MS management. The growth of confidence in the referral neurologist is highlighted by Table 10 indicating the sincerity in reporting adherence to the therapy; a statistically significant difference in the McNemar test with continuity correction (*P* = 0.048) is detected at 3 and 12 months.

A constant relationship with the center allows the patients to follow a diagnostic and therapeutic process based on national and international guidelines. The relevance of the neurologist stems clearly from our data, as the role of the general practitioner is considered less important (33% versus 96%) (Table 2). The central role in disease management and fidelity to the referral center are typical of MS management in Italy where the clinical centers provide a total care of the patient (diagnosis, clinical and pharmacological management of symptoms and relapses, workup prescription, management of side effects, referral to other specialists, interpretation of results, and management of intercurrent and extraneurological diseases). It is very likely that the intense involvement in patient care by the center is the main motivation for the high percentage of adherence that we observed.

Nurses specifically trained in the management of MS patients are regarded as equally important (see Table 3) in coping with illness, in patient training, and in drug administration management (50.3%) but not as “support for injections” (Table 5). The main resource to cope with the illness in the first place consider the family as the most important, but doctors, nurses, and the hospital are just after in the belief of patients.

A clear and focused doctor-patient relationship, aimed to control the appearance of “unrealistic optimistic expectations” [24], is important to maintain motivation and thus adherence in patients with MS. In addition, ongoing training and constant reinforcement of the value of treatment strategies are essential to maintain adherence to treatment. Although other factors such as cognitive [21] or psychiatric disorders [13] may favor patients' forgetfulness of drug administration, positive reinforcement or other strategies including a proper management of treatment expectations should be used to promote adherence, reinforce perceived effectiveness, and minimize adverse events [9, 21, 24].

## Figures and Tables

**Table 1:** Demographic data.

Sex	Females 198 (69.5%)	Males 86 (30.2%)	
Age	Mean 36.1 (10.9 SD)	Min 16.6—max 61.6	
Education	Years 12.3 (3.0 SD)		
	Lower school	3 months: 69 (26.3%)	12 months: 61 (24.6%)
	High school	3 months: 136 (51.9%)	12 months: 134 (54.0%)
	Graduate	3 months: 42 (16.3%)	12 months: 42 (16.5%)
	Other/ND	3 months: 15 (5.7%)	12 months: 12 (4.8%)
Work	Full or partial time	M 72.2%	F 56.0%
	Housewives		F 13.5%
	Student	M 5.1%	F 11.4%
	Retired	M 8.9%	F 2.2%
	Unemployed	M 13.9%	F 12.0%
Social status	Alone	M 5.1%	F 8.0%
	With parents	M 34.2%	F 28.3%
	With others	M 60.7%	F 63.7%
Age at MS onset	31.2 (9.4 SD)	Min 9.0—max 57.5	
Age at diagnosis	34.0 (9.8 SD)	Min 9.7—max 58.1	
Time from diagnosis to therapy starting	Years 1.9 (3.9 SD)		

**Table 2:** “Which health professional is mostly involved in MS management?” (multiple answers possible).

	Three months		Twelve months	
	*N*	%	*N*	%
Neurologist	253	96.56	239	96.37
General doctor	90	34.35	83	33.46
Nurse	77	29.38	65	26.20
Physiotherapist	18	6.87	18	7.25
Other	3	1.14	7	2.82
Home assistant	1	0.38	0	
No one	1	0.38	3	1.20

**Table 3:** “What is your main resource in coping with the illness?”

	Three months		Twelve months	
	*N*	%	*N*	%
Parents				
Low	82	31.29	86	34.67
Medium	46	17.55	47	18.94
High	134	51.13	115	46.36
The family				
Low	21	8.00	37	14.91
Medium	27	10.30	7	2.82
High	214	81.67	204	82.35
Faith/religious belief				
Low	108	41.22	88	35.47
Medium	63	24.05	61	24.59
High	91	34.73	99	39.91
Friends				
Low	99	37.78	79	31.85
Medium	76	28.99	73	29.33
High	87	33.19	96	38.70
Support Groups				
Low	202	77.09	172	69.35
Medium	37	14.11	51	20.56
High	23	8.77	25	10.07
Doctors/nurses				
Low	42	16.02	39	15.72
Medium	44	16.78	54	21.77
High	176	67.16	155	62.49
Hospital Center				
Low	55	20.98	45	18.13
Medium	39	15.26	50	20.15
High	167	63.73	153	61.69

**Table 4:** “Who decided to start therapy?”

	Three months	
	*N*	%
I decided	3	1.14
I decided after discussing it with the neurologist	29	11.06
I together with my neurologist	92	35.11
My neurologist, even considering my opinion	75	28.62
My neurologist	59	22.51
ND	4	1.52

(This question is only on the 3-month questionnaire).

**Table 5:** Main factors in the choice of MS Therapy.

	Three months		Twelve months	
	*N*	%	*N*	%
The drug's mechanism of action				
Low	36	13.9	25	10.4
Medium	94	36.3	86	35.7
High	129	49.8	130	53.9
The drug reduces the relapses				
Low	22	8.5	14	5.8
Medium	71	27.4	72	29.9
High	166	64.1	155	64.3
The drug slows the disease progression				
Low	24	9.3	14	5.8
Medium	61	23.6	69	28.6
High	174	67.2	158	65.6
The drug produces fewer antibodies				
Low	93	35.9	69	28.6
Medium	125	48.3	134	55.6
High	41	15.8	38	15.8
The drug is well known				
Low	44	17.0	40	16.6
Medium	113	43.6	102	42.3
High	102	39.4	99	41.1
The drug has few side effects				
Low	29	11.2	21	8.7
Medium	97	37.5	112	46.5
High	133	51.4	108	44.8
The drug improves the MRI				
Low	61	23.6	36	14.9
Medium	87	33.6	96	39.8
High	111	42.9	109	45.2
The nurse support for injections				
Low	146	56.4	121	50.2
Medium	63	24.3	82	34.0
High	50	19.3	38	15.8
Information support of the company producing the drug				
Low	104	40.2	86	35.7
Medium	104	40.2	113	46.9
High	51	19.7	42	17.4
The intramuscular or subcutaneous mode of administration				
Low	55	21.2	47	19.5
Medium	118	45.6	122	50.6
High	86	33.2	72	29.9
The ability to let by myself injections				
Low	41	15.8	27	11.2
Medium	79	30.5	84	34.9
High	139	53.7	130	53.9
The availability of an autoinjector				
Low	70	27.0	42	17.4
Medium	73	28.2	79	32.8
High	116	44.8	120	49.8
How many times a week should I take the drug?				
Low	49	18.9	35	14.5
Medium	114	44.0	103	42.7
High	96	37.1	103	42.7
The independence that a treatment can give				
Low	33	12.7	28	11.6
Medium	71	27.4	87	36.1
High	155	59.8	126	52.3

**Table 6:** “How often is the medication taken according to neurologist prescription?”

	Three months		Twelve months	
	*N*	%	*N*	%
Always	255	97.32	233	93.95
The dose is reduced	2	0.76	2	0.80
The dose is increased	0	0.00	1	0.40
The number of injections is reduced	2	0.76	6	2.41
ND	3	1.14	6	2.41

**Table 7:** Cause of change in the number or dosage of injections.

	3 months		12 months	
	*N*	%	*N*	%
Dosage inconvenient or difficult	2	1.7	1	0.9
Forgot the administration	17	14.5	20	17.4
The injection generated anxiety	14	12.0	15	13.0
“I did not feel the need to do all injections”	1	0.8	3	2.6
Tired of injections	11	9.4	9	7.8
No one could do the injection	4	3.4	1	0.9
Skin reactions	6	5.1	8	6.9
Pain at the injection site	12	10.3	12	10.4
Flu-like syndrome	12	10.3	5	4.3
Depression	5	4.3	5	4.3
Headache	3	2.6	5	4.3
Fatigue	10	8.5	9	7.8
Weakness	9	7.7	8	6.9
“I did not get therapy”	3	2.6	1	0.9
Not sure of the benefits	3	2.6	4	3.5
Pregnant or plan to become pregnant	0	0.0	1	0.9
More	5	4.3	8	6.9

**Table 8:** “Would you tell your neurologist you did not follow his prescriptions?”

	Three months				Twelve months			
	*N*	%	% M	% F	*N*	%	% M	% F
Yes	220	83,96	76.3	87.8	219	88,30	84.5	92.9
No	40	15,26	23.7	12.2	24	9,67	15.5	7.1
ND	2	0,76			5	2,01		

**Table 9:** “Forgotten, by choice or by accident, a few injections in the last 4 weeks?” (∗).

	Three months		Twelve months	
	*N*	%	*N*	%
Yes	37	14.12	40	16.12
No	225	85.87	199	80.24
ND	—	—	9	3.62

(∗) “few” intended as 1 dose/month of interferon *β*1a-im, 1–3 doses/month of other interferon or glatiramer acetates.

**Table 10:** “Decided to modify the prescribed dose in the last 4 weeks?”

	Three months		Twelve months	
	*N*	%	*N*	%
Yes	7	2.8	10	4.2
No	244	97.2	227	95.8
